# Supplementary material for: Combining multi-dimensional data to identify key genes and pathways in gastric cancer
Source: PeerJ. 2017 Jun 6;5:e3385. doi: 10.7717/peerj.3385 (PMC5463969; doi:10.7717/peerj.3385)
Supplement: Supplemental Information 1 [file peerj-05-3385-s005.docx]

#Install bioconductor and limma package

source("http://bioconductor.org/biocLite.R")

biocLite(‘limma’)

#Launch limma

library(limma)

options(stringsAsFactors = F)

#Read the data

data <- read.csv("XXX.csv",row.names=1)

data <- apply(data,c(1,2),as.numeric)

data <- as.matrix(data)

#Remove 0

datMat.log<- log2(pmax(data,min(data[data!=0])))

#Cluster the samples, 0 represents normal and 1 represents tumor; N is the number of samples

myCluster <- c(rep(0,N),rep(1,N))

#Design the model

myDesign <- model.matrix(~factor(myCluster))

# Fitting a linear model

fit <- lmFit(object = t(datMat.log),design = myDesign)

#Apply empirical Bayes smoothing

fit <- eBayes(fit)

# Show statistics for the top genes/miRNAs/methlations

degTable <- topTable(fit,number = Inf)

#Select significant genes/miRNAs/methylation-sites

deg <- degTable[degTable[,5]<0.01,]

deg1 <- deg[deg[,1]< -1,]

deg2 <- deg[deg[,1]>1,]

deg <- rbind(deg1,deg2)
