# Supplementary material for: Combining multi-dimensional data to identify key genes and pathways in gastric cancer
Source: PeerJ. 2017 Jun 6;5:e3385. doi: 10.7717/peerj.3385 (PMC5463969; doi:10.7717/peerj.3385)
Supplement: Supplemental Information 2 [file peerj-05-3385-s006.docx]

#Read the data

data1 <- read.csv("XXX1.csv",row.names=1)

data2 <- read.csv (“XXX2.csv”,row.names=1)

m <- ncol(data1)

n <- ncol(data2)

#calculate r value

result1 <- matrix(nrow=m,ncol=n)

for (i in (1:m)){

for(j in (1:n)){

result[i,j] = cor(data1[,i],data2[,j],method="*pearson*")

}

}

row.names(result1) <- colnames(data1)

colnames(result1) <- colnames(data2)

library(reshape2)

Cor1 <- melt(result1,id.vars=c("colnames(result1)", "rownames(result1)"), value.name= "cor")

#Calculate *P*-value

result2 <- NULL

for(i in (1:m)){

p <- NULL

for (j in (1:n)){

a <- cor.test( data1[,i], data2[,j], method="*pearson*")

p <- c(p, a$p.value)

}

result2 <- cbind(result2, p)

}

result2 <- t(result2)

row.names(result2) <- colnames(data1)

colnames(result2) <- colnames(data2)

Cor2 <- melt(result1,id.vars=c("colnames(result2)", "rownames(result2)"), value.name= "*P*-value")

#Combine data

Cor <- cbind(Cor1, Cor2[,3])
